# The Online Market for Healthcare Self‐Testing Kits: Benefits, Risks and Media Representation in the United Kingdom

**DOI:** 10.1002/puh2.70342

**Published:** 2026-08-11

**Authors:** Jasmine Lee, Sebastian S. Fuller, Suneeta Soni, Emma Harding‐Esch

**Affiliations:** ^1^ Lancaster University Bailrigg UK; ^2^ Nuffield Department of Medicine University of Oxford Oxford UK; ^3^ University Hospital Sussex Royal Sussex County Hospital Brighton UK; ^4^ London School of Hygiene & Tropical Medicine London UK

**Keywords:** media representation, online market, self‐testing kits, United Kingdom

## Abstract

**Introduction:**

The UK private healthcare self‐testing market has grown quickly, especially since the COVID‐19 pandemic. Self‐tests offer convenience and autonomy, but concerns persist about their accuracy, regulation, oversight and follow‐up care. Media coverage shapes public views, but systematic analysis is lacking. Our aims were to examine UK media coverage of private self‐testing kits, identifying key themes, reported benefits and risks and overall media attitudes.

**Methods:**

A search of online media articles published between 2019 and 2023 was conducted using Google. Eligible articles discussed privately purchased self‐testing kits, were in English, and were free to access. Articles were grouped by health area, and thematic analysis found key patterns.

**Results:**

Fifty‐nine articles met inclusion criteria. Themes that were described as positive included autonomy, accessibility and use alongside the National Health Service (NHS). Negative themes included concerns about accuracy, the use of inappropriate biomarkers, insufficient regulation, lack of follow‐up, the risk of misinterpretation, psychological distress and an increased burden on the NHS. Media reports noted a lack of standardisation compared with NHS pathways.

**Conclusion:**

UK media coverage was predominantly critical of self‐tests, although benefits such as accessibility and autonomy were recognised. Media representations are influenced by journalistic framing, selective sourcing and broader tendencies toward risk‐focused reporting. Future research should explore how this influences public behaviour, engagement with self‐testing technologies and subsequent healthcare utilisation.

## Graphical abstract

UK online media coverage (2019–2023, 59 articles) of private self‐testing kits was predominantly critical. Recognised benefits of autonomy and accessibility were outweighed by concerns over accuracy, inadequate regulation, poor follow‐up and psychological harm. Journalistic framing and selective sourcing likely shape public perception and health‐seeking behaviour around self‐testing.

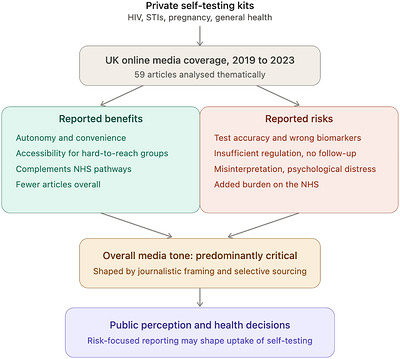

## 1 Introduction

In recent years, the United Kingdom (UK) market for healthcare self‐testing kits has expanded, accelerated by the COVID‐19 pandemic [1]. COVID‐19 testing normalised self‐testing and increased consumer confidence [1, 2]. Self‐testing encompasses a wide range of diagnostic products addressing different health conditions, including infectious diseases (e.g., HIV and other sexually transmitted infections [STIs]), reproductive health (e.g., pregnancy and fertility testing) and general health monitoring (e.g., glucose testing or vitamin levels). For example, self‐tests for pregnancy and certain infectious diseases are supported by strong evidence and established regulatory frameworks, with clear pathways for follow‐up care [1, 3]. In contrast, some tests, such as broad health screening panels, hormone tests or tests based on non‐recommended biomarkers, have a more limited evidence base supporting their clinical utility and may not align with national screening guidelines [4]. The populations using self‐tests are also diverse, including individuals seeking routine monitoring or reassurance, as well as those facing barriers to accessing traditional healthcare services, such as stigma, access to traditional healthcare settings or long waiting times [5].

The UK National Health Service (NHS) has increasingly incorporated digital healthcare services, including remote consultations, online triage systems and limited forms of self‐testing [1]. Examples include NHS‐supported self‐testing during the COVID‐19 pandemic and self‐sampling services for STIs, where individuals collect samples at home and return them for laboratory analysis [6, 7]. These services are typically embedded within established care pathways and include mechanisms for results interpretation and follow‐up care [6]. Long waiting times, regional variation in service provision, and barriers related to stigma or convenience may limit access to traditional NHS pathways [8]. Private providers have emerged to address these gaps, offering rapid, direct‐to‐consumer testing services that emphasise speed, autonomy and convenience [1, 3]. Self‐test providers include both online platforms and tests sold through pharmacies and retail outlets, although they may operate outside standardised clinical pathways and with varying levels of clinical oversight [9].

Not all private tests are clinically appropriate or beneficial. The UK National Screening Committee (NSC) recommends that screening tests should only be used when benefits outweigh harms, tests are validated, and appropriate follow‐up is available [4, 10]. The Royal College of General Practitioners (RCGP) and the British Association for Sexual Health and HIV (BASHH) have raised concerns about diagnostic accuracy, validation, follow‐up and the burden on primary care that may result from direct‐to‐consumer or self‐tests [6, 11, 12]. For tests with limited validation or unclear clinical relevance, risks may include false reassurance, unnecessary anxiety, inappropriate treatment decisions and increased demand on NHS services [4].

The media, therefore, plays an important role in shaping public understanding of self‐testing and offers insights into how benefits, risks and uncertainties are communicated [13]. However, media coverage may not consistently distinguish between different types of self‐tests or reflect the strength of the underlying evidence [13].

Given the diversity of self‐testing technologies and their varying levels of evidence and regulation, it is important to understand how these tests are represented in the media. This study involved a scoping review of UK online media articles discussing private healthcare self‐testing kits available for direct purchase by the general public. The main objectives were to identify which private self‐tests are covered, and analyse recurring themes, reported benefits and risks, and overall media attitudes.

## 2 Methods

### 2.1 Study Design

We conducted a scoping review, analysing UK media articles on private healthcare self‐testing. ‘Self‐test’ included any test conducted by an individual, with results either interpreted at home or via laboratory analysis [7]. This approach explored media narratives, perceived benefits, risks and societal implications.

### 2.2 Search Strategy

**FIGURE 1:**
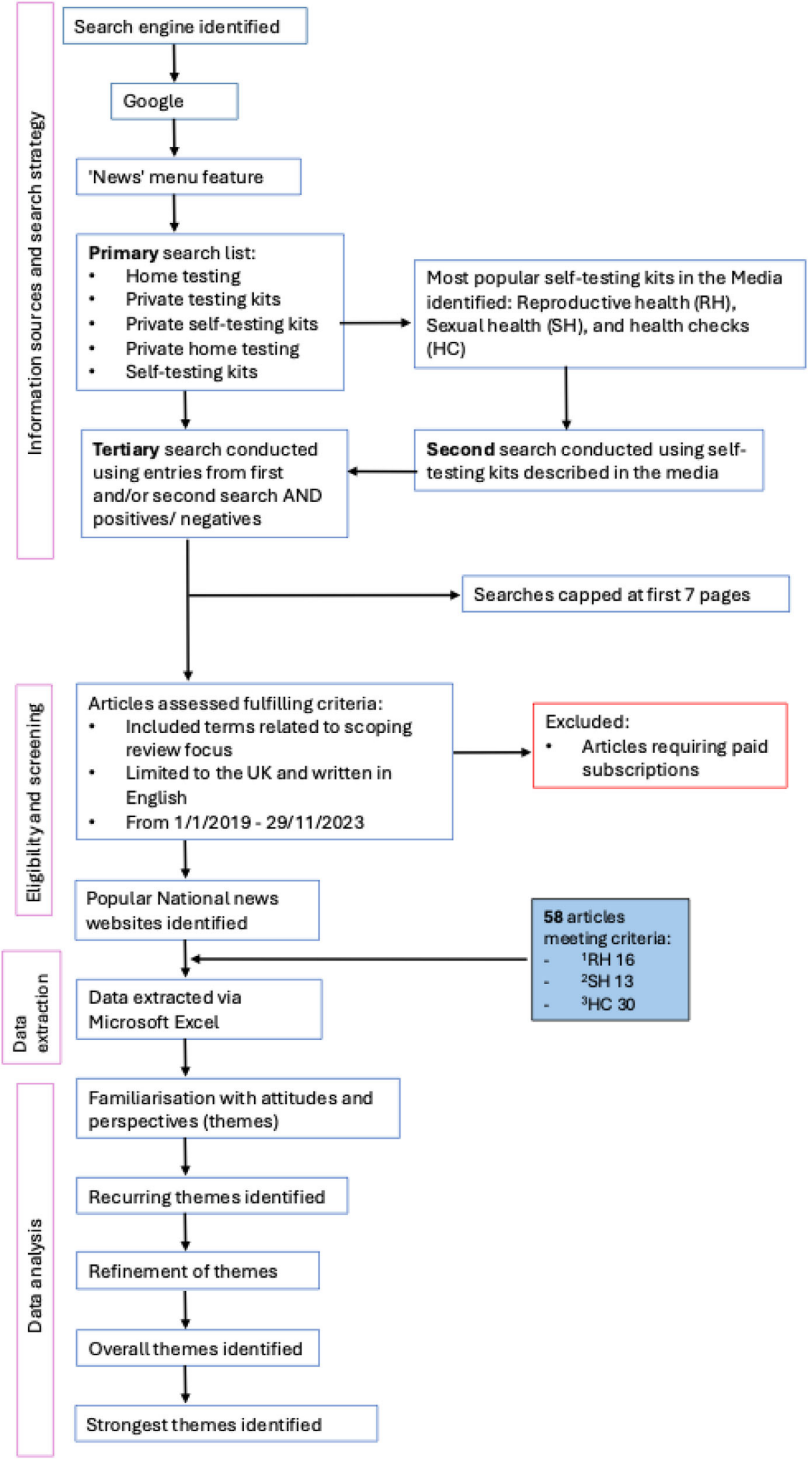
Flow chart of article identification, screening and inclusion process.

Figure 1 summarises the article identification, screening and inclusion process. Media articles were identified using Google News (October–November 2023), employing broad terms to find widely accessed articles, such as ‘home testing’, ‘private testing kits’ and ‘private self‐testing kits’. Google News was selected as the primary search platform as it aggregates an extensive range of widely accessed UK news sources and reflects content most likely to be encountered by the general public. This aligns with the study aims of examining media representations as experienced by typical users. However, as Google search results may be influenced by personalisation algorithms based on user location, search history and browsing behaviour, efforts were made to standardise the search approach, including using a consistent browser setup and avoiding personalised accounts. A secondary search used the following self‐testing kit categories: reproductive health, STIs and general health checks; with fertility and menopause grouped under reproductive health. A third search included terms for positive and negative reporting. Screening was limited to the first seven pages of results for each term to reflect typical user engagement with search outputs. Supporting Information 1 lists all search terms.

### 2.3 Eligibility Criteria

Article screening was conducted using predefined inclusion and exclusion criteria. Where uncertainty arose, decisions were discussed among the research team to ensure consistent application of criteria. Articles were included if they were published between January 2019 and November 2023, English‐language, UK‐based and publicly accessible without subscription. Eligible articles discussed privately purchased self‐testing kits and included opinion pieces or news reports discussing advantages, disadvantages or other aspects of these products. Articles were excluded if they required paid access, published outside the study period, were not UK‐based, were not written in English, focused exclusively on NHS testing, did not primarily discuss private self‐testing kits or were duplicate reports.

### 2.4 Data Extraction

Relevant data were extracted and tabulated in Microsoft Excel Version 16.84 (24041420). Data extraction focused on segments of text within each article that expressed opinions, interpretations or claims about private self‐testing kits. These included statements describing perceived benefits, risks, regulatory concerns, user experiences and expert commentary. Relevant excerpts were systematically identified and recorded to capture how self‐testing was portrayed in the media. Extracted data were documented systematically to maintain an audit trail, supporting transparency in how themes were developed and refined.

### 2.5 Data Analysis

Inductive thematic analysis identified perspectives and attitudes in the media [14]. After familiarisation with the dataset, initial codes were generated to capture key patterns. Codes were reviewed and grouped into broader patterns, which were refined into the ‘strongest themes’ on the basis of frequency and prominence. Initial coding and thematic analysis were conducted by a single researcher. To enhance validity and reduce subjective bias, coding decisions and emerging themes were refined through discussions with co‐authors throughout the analysis process. This iterative approach resulted in our final agreed list of themes, ensuring consistency and robustness of interpretation.

## 3 Results

### 3.1 Categories of Self‐Testing Kits

Fifty‐nine articles met the criteria, covering various private self‐testing kits. Articles were grouped into three main health categories:

1. Reproductive health: menopause and fertility‐related tests (*n* = 16);
2. STIs: including *Ureaplasma* species (spp.), *Mycoplasma* spp. and other infections (*n* = 13);
3. General health checks: covering markers, such as blood counts, liver and renal function, lipid profiles, vitamin deficiencies, cancer, thyroid function, and inflammatory markers (*n* = 30).

Where relevant, articles reporting on both menopause and fertility were grouped under reproductive health.

### 3.2 Overview of Themes

Table 1 summarises the strongest themes identified across each health category and the number of supporting articles. Table 2 presents example quotes supporting each theme.

**TABLE 1:** Summary of the strongest themes identified across each health category, listing the number of supporting articles and overall impression of the theme.

Themes	Reproductive health (*n* = 16)	Sexual health (*n* = 13)	General health (*n* = 30)	Total number of articles reporting on the theme
**Convenience and accessibility**	4	5	9	18
**Autonomy and empowerment**	6	2	10	18
**Complementary to NHS**	4	—	—	4
**Diagnostic accuracy and reliability concerns**	—	4	11	15
**Inappropriate biomarkers tested**	11	9	12	32
**Lack of regulation and oversight**	5	7	10	22
**Misinterpretation of results**	9	—	10	19
**Aftercare decisions**	9	—	11	20
**Lack of follow‐up**	—	7	5	12
**NHS burden**	6	—	13	19
**Psychological impact**	7	—	16	23
**Public health impact**	—	7	—	7

Abbreviation: NHS, National Health Service.

**TABLE 2:** Example quotes supporting each theme.

Theme	Example quote, followed by the reference in brackets
**Convenience and accessibility**	‘Convenient and accessible options for monitoring their health at home’ [5]
**Autonomy and empowerment**	‘Take their health into their own hands at an affordable price’ [15]
**Complementary to NHS**	‘Knowing your FSH levels and seeking medical help’ [16]
**Diagnostic accuracy and reliability concerns**	‘Unnecessary testing, especially if sensitivity is high, can lead to many false positive results’ [9]
**Inappropriate biomarkers tested**	‘Inappropriate infections were tested for’ [17]
**Lack of regulation and oversight**	‘Warned it is not yet clear how these tests are being regulated nationwide’ [9]
**Lack of follow‐up**	*‘*Lack of follow‐up and continuity of care’ [5]
**Public health impact**	‘It can have personal consequences, and also wider public health consequences’ [18]
**Misinterpretation of results**	*‘There is a risk that results might be misinterpreted or be misleading’* [11]
**Psychological impact**	‘Results could cause anxiety and confusion’ [19]
**Aftercare decisions**	‘Patients may not know how to properly interpret results, or safely and appropriately act on them’ [15]
**NHS burden**	[tests results are] ‘leaving an already overworked HS to follow up ‘abnormal results’ [20]

Abbreviations: FSH, follicle‐stimulating hormone; NHS, National Health Service.

The strongest themes identified fit two domains as follows:

1. Perceived benefits—focusing on accessibility, convenience and autonomy.
2. Perceived risks—accuracy, regulation, psychological impact and consequences for healthcare services.

Although benefits such as accessibility, autonomy and convenience were consistently acknowledged, the volume, depth and framing of risk‐related themes (particularly concerns around accuracy, regulation and psychological harm) suggest that coverage was more critical than supportive. For example, the most frequent risk‐related theme, ‘inappropriate biomarkers tested’, was expressed in 32 articles, whereas the most reported benefit‐related theme, ‘autonomy and empowerment’, appeared in 18 articles [15, 17].

### 3.3 Perceived Benefits

Media coverage frequently described private self‐testing kits as improving accessibility and convenience across all health categories. Articles emphasised the ability to conduct these tests at home, avoiding appointments and reducing waiting times. For example, some reports described self‐testing as ‘convenient and accessible options for monitoring their health at home’ [5, 21].

Autonomy and empowerment were also commonly reported, especially in reproductive health contexts. Self‐testing was framed as enabling individuals to take ‘health into their own hands at an affordable price’ [15], reflecting a broader shift toward consumer‐led healthcare [8, 19]. However, this theme was often presented descriptively rather than critically, with limited discussion of potential limitations.

Within reproductive health, self‐tests were discussed as complementary to NHS services, serving as a preliminary step to seeking professional care rather than as a substitution.

### 3.4 Perceived Risks

As shown in Table 1, concerns relating to testing for inappropriate biological markers and lack of regulation were discussed throughout all health categories.

Concerns regarding diagnostic accuracy and reliability were among the most prominent, particularly sexual health and general health testing. Articles frequently highlighted the potential for false positive or negative results, emphasising uncertainty around the clinical validity of some tests [9, 22]. A closely related and highly prevalent theme, ‘inappropriate biological markers tested’, appeared in articles emphasising that some tests targeted biomarkers not supported by national screening guidelines and raised concerns about misleading results and inappropriate use [11, 17].

The potential for misinterpretation of results was also commonly discussed, particularly in articles focused on reproductive health and general health, reporting concerns that individuals may struggle to interpret results without professional guidance [20]. This was closely linked to impact on aftercare and clinical decision‐making, with articles suggesting unclear or misleading results could influence health behaviours, including delay in seeking advice or inappropriate self‐management [15, 19]. Lack of follow‐up support was highlighted within sexual health and general health categories, with providers offering little or no support for interpreting results, leaving users without guidance [5, 17]. These articles frequently warned readers that unclear or worrying private test results led consumers to seek NHS advice, increasing demand on NHS services [20, 23].

Negative psychological impact as a result of self‐testing kits was evident within the reproductive health and general health categories. Concerns with consumers receiving results without professional help were reported to cause anxiety, confusion or distress; this was particularly discussed within general health checks [9, 19].

Broader public health implications were highlighted most prominently in sexual health. Media coverage raised concerns that inaccurate or unreported results could undermine disease surveillance and delay diagnosis [18].

## 4 Discussion

### 4.1 Overview and Key Findings

Overall, media coverage portrays a mixed but predominantly critical narrative. Although benefits, such as autonomy, convenience and accessibility, are acknowledged, risk‐related themes, including concerns about reliability, accuracy, negative psychological impact and strain on NHS healthcare services, are more frequently reported and discussed in greater depth.

The extent to which media narratives aligned with scientific evidence was not systematically evaluated within this study. Our findings reflect media representations of self‐testing rather than direct scientific evaluation of the tests themselves. The perspectives identified in this analysis were drawn from how journalists presented the views of experts, industry representatives and commentators on self‐testing within media articles and therefore may not fully reflect the underlying clinical evidence base. Instead, they represent how these issues are communicated to the public.

### 4.2 Public Perceptions: Autonomy, Empowerment and Convenience

A key theme emerging from media coverage was the framing of self‐testing kits as a means of increasing consumer autonomy, allowing individuals to ‘take health into their own hands’ [3, 15, 21]. Self‐testing offers practical advances such as convenience, privacy and flexibility and may improve access for individuals who face barriers to traditional NHS services [3, 24, 25]. However, these benefits are often presented alongside cautionary commentary. The potential for misinterpretation, lack of professional guidance and reduced engagement with healthcare providers introduces tension between autonomy and safety [5]. Thus, empowerment is frequently portrayed as conditional rather than unequivocally beneficial.

### 4.3 Integration With NHS Services and System Impact

Media narratives suggest that self‐testing may complement existing NHS care services by encouraging early engagement and reducing unnecessary appointments [9, 16, 24]. However, the lack of integration with established care pathways creates challenges. Inconsistent quality, unclear results and limited follow‐up often shift the burden of interpretation and management onto NHS healthcare professionals [4, 26]. Media reporting frequently highlights this as a concern, reinforcing the perception that self‐testing may contribute to, rather than alleviate, system strain.

### 4.4 Regulation, Validation and Diagnostic Accuracy

Conformité Européenne (CE) or UK Conformity Assessed (UKCA) markings are required for tests to be sold within the European Economic Area or UK; such certification primarily confirms that the device measures the parameter it claims to assess, rather than validating its clinical reliability [3, 6, 27]. Obtaining CE or UKCA marking for self‐testing kits is relatively straightforward; many of these tests are often not sufficiently validated and do not undergo the level of scrutiny applied to NHS tests accredited by the United Kingdom Accreditation Services (UKAS) or overseen by the Care Quality Commission (CQC) [1, 6, 11, 28]. These limitations are especially concerning for infectious or chronic conditions, where inaccurate results may compromise patient safety and public health [22].

The absence of independent verification stated in the media underscores the potential for false‐positive results, which may precipitate unnecessary anxiety, inappropriate treatment decisions and suboptimal antimicrobial stewardship, as well as false‐negative results, which can delay diagnosis, obscure infection and increase the risk of onward transmission [3, 6, 11]. This point is exemplified by a BBC news article from 2025, which highlighted findings from researchers at the University of Birmingham who found that evidence supporting many manufacturers’ accuracy claims was largely unavailable [3].

### 4.5 Clinical and Ethical Implications

Certain self‐tests do not align with UK guidelines, the National Institute for Health and Care Excellence (NICE) uses evidence‐based medicine methodologies to define appropriate testing criteria and parameters in the development of clinical guidelines [29]. However, many private tests deviate from these standards, posing risks to both individuals and healthcare systems [9].

A particular area of focus was testing for inappropriate biomarkers and the clinical and ethical implications. Multiplex assays, increasingly used in infection screening, can detect organisms that are not clinically significant [6, 11]. Private testing for certain infections not routinely screened by the NHS may lead to inappropriate treatment, increased antibiotic use and diminished antimicrobial stewardship, raising ethical concerns for public health [6, 11]. For example, isolated follicle‐stimulating hormone (FSH) testing for menopause is not routinely recommended by clinical guidelines, the Royal College of Obstetricians and Gynaecologists (RCOG) or the British Menopause Society (BMS) and may mislead users about fertility or menopause status, leading to inappropriate decisions, unplanned pregnancies or delayed care [19, 30]. Other inappropriate biomarkers include prostate‐specific antigen (PSA) for prostate cancer, which is not recommended for screening due to the influence of multiple lifestyles and psychological factors influencing PSA results [4, 10, 26]. Self‐testing without guidance may increase anxiety or result in unnecessary investigations, which can be costly, invasive and potentially widen health inequalities among patients unable to afford such procedures and disproportionately affect vulnerable groups, raising concerns about equity and informed decision‐making [1, 4, 10].

These issues contribute to a boarder narrative surrounding patient safety, informed decision‐making and equity [4]. Without adequate regulation and guidance, self‐testing may exacerbate health inequalities and place individuals in positions where they must interpret complex medical information without support.

### 4.6 Public Health and Data Implications

The implications of private self‐testing kits pose challenges for public health surveillance and data governance, as results are often not reported to national databases [1, 6, 11]. Individuals who test privately and do not seek confirmatory testing through the NHS remain absent from official records, potentially leading to underestimation or misrepresentation of disease burden [6, 11]. This is particularly relevant for STIs and antimicrobial resistance, where timely reporting and contact tracing are essential for public health control [11, 31]. Without oversight, fragmented data streams risk undermining public trust and effective health surveillance [6, 11].

Expansion of private diagnostics also raises ethical concerns about data privacy. Providers may not follow NHS data standards and could share personal data with third parties without adequate consent, highlighting gaps in current regulations [4, 6, 11].

Inequalities in access and health literacy may widen disparities in which uses private self‐tests [1, 3]. Misleading advertising and a lack of support can increase unnecessary NHS utilisation and strain resources [4, 10].

These challenges highlight the urgent need for unified national frameworks for validating, reporting and handling self‐testing data, ensuring safe, equitable and privacy‐protected access [6, 11].

### 4.7 Media Framing, Bias and Sources of Perception

Although both benefits and risks were represented in media reports, their framing was weighted toward highlighting potential harms and uncertainties, highlighting questions of safety, reliability and broader implications. The predominance of risk‐focused reporting observed in this study aligns with broader patterns in health journalism, where negative or cautionary narratives are often prioritised. Media outlets tend to emphasise potential harm, uncertainty and controversy, which may amplify perceptions of risk even when benefits are present [32].

The perspectives presented in media coverage originate from a range of sources, including healthcare professionals, academic experts, regulatory bodies, industry representatives and individual patient experiences. However, these viewpoints can be selectively reported and framed by journalists, which may introduce bias or emphasise particular narratives [32]. Media articles do not consistently distinguish between evidence‐based statements and opinion, and the importance given to a specific voice may shape the overall tone of reporting.

As a result, media representations do not necessarily provide a comprehensive or fully accurate reflection of the scientific landscape, but rather a curated and interpreted version shaped by narrative, accessibility and audience engagement [32]. This has implications for public understanding. Although the emphasis on risks may support cautious and informed decision‐making, it may also limit awareness of legitimate benefits and appropriate use cases.

### 4.8 Alignment With Scientific Evidence

Comparison with existing scientific literature suggests partial alignment between media narratives and evidence, but also important discrepancies. Concerns frequently highlighted in the media, such as issues with diagnostic accuracy, lack of validation and insufficient regulation, are supported by emerging research demonstrating variability in test performance and oversight within the private self‐testing market. However, media coverage may underrepresent contexts in which self‐testing is effective and well‐established, such as pregnancy testing or certain regulated infectious disease tests, where evidence supports appropriate use when tests are validated and accompanied by clear guidance [1, 11]. Furthermore, media reporting often does not distinguish clearly between different types of self‐tests, presenting the market as a relatively uniform category despite substantial variation in clinical validity, intended use and regulatory standards [7]. This lack of nuance may contribute to public misunderstanding and oversimplification of both risks and benefits.

Overemphasis on risks may discourage appropriate use of validated self‐tests, whereas insufficient explanation of limitations may lead to misuse or overreliance on less reliable tests. Media framing may, therefore, influence public awareness, uptake and follow‐up behaviours, including whether individuals seek professional advice after testing.

### 4.9 Study Limitations

This study had several limitations. We only searched Google News as the source for media articles. Although additional databases or search engines could have been used to validate results, the study prioritised capturing publicly accessible and commonly encountered media content. This approach may have limited completeness but reflects real‐world exposure to media reporting. Other factors affecting comprehensiveness of the data were subscription requirements, which restricted access to some media sources, and search terms which may not have captured all relevant articles. Only English‐language UK articles were included, potentially limiting generalisability. The review covered only a defined time period and may not reflect recent developments in test quality and oversight or evolving attitudes. Despite these limitations, this study provides key insights into media representation and highlights areas needing regulatory oversight, education and support.

### 4.10 Policy and Future Recommendations

Addressing private self‐testing challenges requires coordinated policy to ensure diagnostic reliability, public health protection and patient autonomy. This review highlights the urgent need for comprehensive regulatory alignment between private providers and the NHS [1, 6, 11]. Clear national regulatory standards are needed so that all self‐testing kits meet validation criteria before market entry [6, 12]. Stricter regulations and oversight by bodies, such as the Advertising Standards Authority (ASA) and the Medicines and Healthcare products Regulatory Agency (MHRA), can prevent misleading claims and improve consumer safety [3, 18]. Integration of private self‐testing data into national health information systems, with secure and ethical data‐sharing, would improve disease surveillance and public health modelling [6, 33]. Lessons from the NHS COVID‐19 ‘Test and Trace’ programme highlight the importance and challenges of self‐reporting systems [33].

Public and professional education are also essential to address risks of misinterpretation. Health literacy campaigns can inform test providers and users about expected standards and warn against poor practice [6, 11]. Clear guidance on correct use and transparent communication about accuracy and limitations can help users make informed decisions and reduce distress, especially for those with lower health literacy [1]. Ethical frameworks should govern advertising, data use and post‐test support.

Finally, closer integration between the public and private sectors is needed to align private self‐testing with NHS standards and reduce strain on NHS services. Enhanced support services could help manage increased demand [3].

## 5 Conclusion

In conclusion, UK media coverage of private self‐testing kits presented a mixed but predominantly critical narrative. Although benefits, such as accessibility and autonomy, were recognised, media reporting more frequently emphasised risks, particularly regarding accuracy, regulation and healthcare impact. Although the focus on risks was justified when reporting on scientifically supported limitations of some self‐tests, for example, those testing for inappropriate biomarkers, examples of appropriate self‐testing practice, such as pregnancy tests, were not highlighted. These findings highlight that media representations are shaped not only by available evidence but also by journalistic framing, selective sourcing and broader tendencies toward risk‐focused reporting. Although such reporting plays an important role in raising awareness of potential harms, a more proportionate and nuanced representation of benefits and risks of different self‐testing options may better support patients with informed decision‐making. Future research should explore how media framing influences public behaviour, engagement with self‐testing technologies and subsequent healthcare utilisation.

## Author Contributions

**Jasmine Lee**: writing – original draft, data curation, investigation, formal analysis, resources. **Suneeta Soni**: conceptualisation, writing – review and editing, methodology, supervision. **Sebastian S. Fuller**: writing – review and editing, formal analysis, supervision. **Emma Harding‐Esch**: conceptualisation, writing – review & editing, methodology, supervision.

## Funding

The authors have nothing to report.

## Ethics Statement

This study analysed publicly available media articles and did not involve human participants, personal data or interventions. In accordance with UK Health Research Authority guidance, research based solely on publicly accessible documents does not require ethical approval. All articles included were free to access without subscription, and no identifiable personal information was collected, analysed or reported. Therefore, ethics approval and participant consent were not required.

## Consent

Patient consent was not required for this study as it did not involve human participants, patient data or identifiable personal information.

## Conflicts of Interest

The authors declare no conflicts of interest.

## Permission to Reproduce Material From Other Sources

No copyrighted material has been used from other sources. All cited material has been appropriately referenced. Where direct quotations from media articles are included, they are reproduced under fair dealing provisions for the purposes of academic critique and review.

## Ethics and Integration Policies

The authors recognise that research addressing public health challenges must adhere to the highest standards of ethical conduct and research integrity, given its potential implications for patient safety, health equity, public trust, and healthcare policy. This study was conducted in accordance with principles of transparency, methodological rigour, and responsible scholarship to ensure that findings are accurate, balanced, and evidence‐informed.

As the research involved analysis of publicly accessible media content and did not include human participants, identifiable personal data, or clinical intervention, formal ethical approval was not required, in‐line with guidance from the UK Health Research Authority.

All sources have been appropriately cited, and media quotations are reproduced accurately for the purposes of academic critique and review. The authors confirm that the work is original, that no data have been fabricated, and that the analysis was conducted independently and without external influence.

Maintaining rigorous ethical standards is essential in public health research to safeguard credibility, inform responsible policy development, and protect public confidence in health communication and healthcare systems.

## Supporting information




**Supporting File 1**: Shows all search terms used.

## Data Availability

The data supporting the findings of this study are derived from publicly available online media articles published between January 2019 and November 2023. Extracted and analysed data are available from the corresponding author upon reasonable request.
