# Supplementary material for: The Online Market for Healthcare Self‐Testing Kits: Benefits, Risks and Media Representation in the United Kingdom
Source: Public Health Chall. 2026 Aug 11;5(3):e70342. doi: 10.1002/puh2.70342 (PMC13460401; doi:10.1002/puh2.70342)
Supplement: Supplementary file 1 [file PUH2-5-e70342-s001.docx]

Supplementary information 1

**Shows all search terms used.**

**Search terms**

Advantages of private home testing

Advantages self testing uk

Concerns of self-testing kits

Cons of home testing kits

Cons of private testing kits

Disadvantages of home self testing kits articles

Disadvantages of private home testing kits articles

Female hormone testing kits

Fertility tests

Health checks home testing

Health checks

Home self testing kits

Home testing Health checks

Home testing kits

Home testing STI

Home testing

Menopause risk of paying for home testing kits

Menopause self-testing kits

Menopause test kits

Menopause testing kits

Menopause tests

Negatives health check private self-testing kits

Negatives home testing health checks

Negatives of menopause testing kits

Negatives of paying for home testing kits UK

Negatives of private home testing for health checks

Negatives of private home testing for STIs

Negatives of private self testing kit reproductive health

Negatives of private sexual health testing kits

Negatives of Private STI testing

Negatives of private testing kits

Negatives of reproductive testing kits

Negatives private home self-testing kits

Paid STI testing

Paid STI tests

Paying for home testing kits

Paying for menopause testing kits

Positives of private menopause self-testing kits

Positives of private self-testing kit reproductive health

Positives of home testing kit health checks

Positives of home testing kits

Positives of home testing STI

Positives of menopause testing kits

Positives of private STI testing kits

Positives of self home testing kits UK

Positives of self-testing kits reproductive health

Positives private self-testing kits health check

Private HIV test kits

Private home STI testing

Private home testing health checks

Private home testing STI

Private home testing

Private self-sampling STI

Private self-sampling

Private self-testing kits health checks

Private self-testing kits STI

Private self-testing kits

Private self-testing STI

Private sexual health tests

Private STI test kits

Private STI test online

Private STI testing

Private test kits

Private testing kits STI

Private testing kits

Private testing kits

Pros of private STI testing kits

Risk of paying for STI testing

Risks of home self-testing kits

Risks of home sexual health tests

Risks of home testing kits

Risks of paid home sexual health tests

Risks of paid home test

Risks of paying for STI testing

Risks of private STI kits

Risks of sexual health tests

Risks of testing ureaplasma

Self-testing kit Health checks

Self-testing kit Menopause

Self-testing kit STI

Self-testing kits

Self-sampling kits STI

Self-sampling kits

Self-sampling kits menopause

Self-testing kits

Sexual health testing kits

Sexually transmitted infections testing

STI testing

Testing kit online
